# The Cost-Effectiveness of Sugemalimab Plus CAPOX in Treating Advanced Gastric Cancer: Analysis from the GEMSTONE-303 Trial

**DOI:** 10.3390/cancers17193171

**Published:** 2025-09-29

**Authors:** Chen-Han Chueh, Wei-Ming Huang, Ming-Yu Hong, Yi-Wen Tsai, Nai-Jung Chiang, Hsiao-Ling Chen

**Affiliations:** 1 Herbert Wertheim School of Public Health and Human Longevity Science, University of California San Diego, La Jolla, CA 92093, USA; 2 Institute of Health and Welfare Policy, College of Medicine, National Yang Ming Chiao Tung University, Taipei 112304, Taiwan; milton850115.md12@nycu.edu.tw (W.-M.H.); ywtsai@nycu.edu.tw (Y.-W.T.); hlc.md11@nycu.edu.tw (H.-L.C.); 3 Medical AI Development Center, Taipei Veterans General Hospital, Taipei 11217, Taiwan; 4 Department of Oncology, Taipei Veterans General Hospital, Taipei 11217, Taiwan; njchiang@vghtpe.gov.tw; 5 School of Medicine, College of Medicine, National Yang Ming Chiao Tung University, Taipei 112304, Taiwan; 6 National Institute of Cancer Research, National Health Research Institutes, Miaoli County 35053, Taiwan

**Keywords:** sugemalimab, capecitabine, oxaliplatin, stomach neoplasms, esophageal neoplasms, cost-effectiveness analysis, decision support techniques, Taiwan

## Abstract

Background/Objectives: Sugemalimab demonstrated clinical efficacy in the GEMSTONE-303 trial, but its cost-effectiveness remains unclear. This study aims to evaluate the cost-effectiveness of sugemalimab in combination with chemotherapy (CAPOX) as a first-line treatment for patients with advanced or metastatic gastric or gastroesophageal junction (G/GEJ) adenocarcinoma, compared to chemotherapy alone, from the perspective of Taiwan’s healthcare payer. Methods: A partitioned survival model was developed to simulate outcomes over a 40-year time horizon, and model parameters were derived from GEMSTONE-303 and the wider literature. Health benefits were measured in quality-adjusted life-years (QALYs), and only direct medical costs were included, with both discounted at an annual rate of 3%. The willingness-to-pay threshold was set at three times the 2024 GDP per capita. Deterministic and probabilistic sensitivity analyses were conducted alongside scenario analyses. Results: Compared to capecitabine and oxaliplatin (CAPOX) alone, adding sugemalimab yielded an incremental gain of 0.39 QALYs at an additional cost of USD 47,020, resulting in an incremental net monetary benefit of −USD 7478. Conclusions: Sugemalimab plus CAPOX is not cost-effective for advanced or metastatic G/GEJ adenocarcinoma from the Taiwan payer’s perspective. Achieving cost-effectiveness would require a 20–30% price reduction for sugemalimab (to USD 1204–USD 1376 per 600 mg), assuming first-line therapy is administered for the median treatment duration observed in the GEMSTONE-303 trial. If reimbursement continued until disease progression, a reduction of approximately 68% would be required (USD 550 per 600 mg).

## Simple Summary

Sugemalimab showed improved survival outcomes for patients with advanced or metastatic gastric cancer in the GEMSTONE-303 trial. However, given its high cost, evaluating its cost-effectiveness is essential. We conducted a model-based economic evaluation to simulate and compare the cost-effectiveness of sugemalimab plus chemotherapy versus chemotherapy alone over 40 years, with the willingness-to-pay for one quality-adjusted life-year set at three times the 2024 GDP per capita. Compared to chemotherapy alone, adding sugemalimab resulted in an incremental gain of 0.39 quality-adjusted life-years at an additional cost of USD 47,020, leading to an incremental net monetary benefit of −USD 7478. Therefore, from the perspective of Taiwan’s healthcare payer, sugemalimab plus chemotherapy is not cost-effective for advanced or metastatic gastric cancer. Achieving cost-effectiveness would require a 20–30% price reduction for sugemalimab (to USD 1204–USD 1376 per 600 mg), assuming reimbursement is limited to the median treatment duration observed in the trial. If reimbursement continued until disease progression, a reduction of approximately 68% would be required (USD 550 per 600 mg).

## 1. Introduction

Gastric cancer is the fifth leading cause of cancer-related death worldwide [1], with a particularly significant impact in certain regions, such as Andean Latin America and East Asia [2,3]. In Asian countries—including Japan, Mongolia, China, Korea, and Taiwan, which represent the most severely affected region globally—gastric cancer accounts for 2.06% to 4.04% of all deaths [2], underscoring its substantial disease burden. For advanced or metastatic gastric cancer, standard chemotherapy, typically a combination of fluoropyrimidine and oxaliplatin, has long been the primary treatment [4]. Recent advances, including the approval of immunotherapies and targeted therapies directed at specific biomarkers such as programmed cell death protein 1 (PD-1), human epidermal growth factor receptor 2 (HER2), and claudin 18.2, have significantly improved overall survival (OS) for these patients [4,5].

Several randomized controlled trials have demonstrated the superior efficacy of immunotherapies targeting PD-1 in advanced gastric cancer, including nivolumab (CheckMate-649, [6,7]), pembrolizumab (KEYNOTE-859, [8,9]), sintilimab (ORIENT-16, [10]), and tislelizumab (RATIONALE-305, [11]). The National Comprehensive Cancer Network recommends administering nivolumab, pembrolizumab, and tislelizumab [4], while the European Society for Medical Oncology suggests nivolumab and pembrolizumab for patients with HER2-negative tumors and a PD-L1 combined positive score (CPS) of 5 or higher [12].

Sugemalimab, a programmed death-ligand 1 (PD-L1) inhibitor, acts on a different component of the PD-1/PD-L1 signaling pathway from other inhibitors and has demonstrated promising results in advanced non-small cell lung cancer [13,14,15], esophageal squamous cell carcinoma [16], and gastric or gastroesophageal junction (G/GEJ) adenocarcinoma [17]. In the GEMSTONE-303 trial, sugemalimab achieved a hazard ratio of 0.66 (95% CI: 0.54–0.81) for progression-free survival (PFS) and 0.75 (95% CI: 0.61–0.92) for OS among patients with advanced or metastatic G/GEJ adenocarcinoma with a PD-L1 CPS ≥ 5 [17]. Sugemalimab is currently approved as a first-line treatment for metastatic non-small-cell lung cancer in the European Union [18], the United Kingdom [19], and China [20]. In China, its indication has recently been expanded to include unresectable, locally advanced, or metastatic G/GEJ adenocarcinoma with PD-L1 expression [21,22].

Taiwan’s National Health Insurance is a universal, single-payer system that covers over 99% of the population (approximately 23 million people). Currently, it only reimburses nivolumab as a first-line treatment for advanced or metastatic G/GEJ adenocarcinoma in patients with HER2-negative tumors and a PD-L1 CPS ≥ 5. Although two recent cost-effectiveness analyses from the perspective of China’s healthcare system concluded that sugemalimab is not cost-effective [23,24], differences in healthcare systems and direct medical costs between China and Taiwan limit the applicability of these findings to the Taiwanese context. Given the limited treatment options and the high costs associated with immunotherapies, particularly within a social insurance framework, it is essential to assess the cost-effectiveness of new therapies in Taiwan. Therefore, this study aims to evaluate the cost-effectiveness of sugemalimab in combination with capecitabine and oxaliplatin (CAPOX) as a first-line treatment for advanced or metastatic G/GEJ adenocarcinoma with a PD-L1 CPS ≥ 5 from the perspective of Taiwan’s healthcare payer. Additionally, this study seeks to recommend an economically justifiable price for sugemalimab.

## 2. Materials and Methods

### 2.1. Health Economic Analysis Plan

We adopted a three-state partitioned survival model comprising progression-free (PF), progressed disease (PD), and death states—a widely accepted approach in oncology—to evaluate the cost-effectiveness of sugemalimab plus CAPOX for patients with advanced or metastatic G/GEJ adenocarcinoma and a PD-L1 CPS ≥ 5. The analysis was conducted from the perspective of Taiwan’s National Health Insurance Administration (NHIA) because innovative cancer drugs are seldom self-paid by patients. Through the NHI and Cancer Drug Fund, the NHIA is the primary payer for cancer treatments and thus determines the market entry and pricing of medicines for approximately 99% of the population. Health outcomes were measured in quality-adjusted life-years (QALYs), and costs included only direct medical expenses reimbursed by the NHIA. Both costs and health outcomes were simulated over a lifetime horizon (40 years) and discounted at an annual rate of 3%. Since Taiwan’s regulatory authorities do not specify a willingness-to-pay (WTP) threshold for advanced cancer treatments, the WTP threshold was set at three times Taiwan’s 2024 GDP per capita (USD 101,949) [25], in line with common practice in oncology cost-effectiveness analyses [23,24,26,27,28,29]. The primary cost-effectiveness metrics were the incremental cost-effectiveness ratio (ICER) and incremental net monetary benefit (INMB). To ensure comprehensiveness and transparency, this study adheres to the Consolidated Health Economic Evaluation Reporting Standards statements (CHEERS; see Table A1) [30,31]. The cost-effectiveness analysis was conducted using the “heemod” package [32] in R (version 4.3.2, Vienna, Austria).

### 2.2. Study Population

The study population comprised treatment-naive patients with unresectable, locally advanced, or metastatic G/GEJ adenocarcinoma. Eligibility criteria were based on the GEMSTONE-303 trial: patients aged 18–75 years with a PD-L1 CPS ≥ 5, an HER2-negative status, and an Eastern Cooperative Oncology Group performance status of 0 or 1 [17]. Additionally, a subgroup analysis was performed for patients with a PD-L1 CPS ≥ 10 to explore potential heterogeneity in cost-effectiveness.

### 2.3. Intervention and Comparator

Treatment protocols mirrored those in the GEMSTONE-303 trial [17]. The intervention group received sugemalimab (1200 mg IV every 3 weeks for up to 24 months) combined with CAPOX (oral capecitabine at 1000 mg/m^2^ twice daily for 14 days and IV oxaliplatin at 130 mg/m^2^ on day 1, every 3 weeks, for up to 6 cycles) [17]. The comparator group received CAPOX alone on the same schedule and at the same dosage for up to 6 cycles [17].

### 2.4. Clinical Efficacy

As individual patient data (IPD) from the GEMSTONE-303 trial were unavailable, we applied Guyot’s method [33] to reconstruct pseudo-IPD by digitizing published Kaplan–Meier curves for PFS and OS using WebPlotDigitizer (version 4.6) [34] and the “IPDfromKM” package [35] in R (version 4.3.2, Vienna, Austria). The accuracy of the reconstructed curves was validated by comparison with the original published data.

For extrapolation to 40 years, pseudo-IPD were fitted to seven parametric survival models in line with the National Institute for Health and Care Excellence recommendations [36]: exponential, Weibull, gamma, log-normal, log-logistic, Gompertz, and generalized gamma. Model selection was based on the Akaike information criterion (AIC), Bayesian information criterion (BIC), visual inspection, and clinical expert opinion (model fit statistics are shown in Table A2). Log-logistic distributions were selected for both groups in the base-case analysis. Detailed model parameters are provided in Table 1.

### 2.5. Treatment-Related Adverse Events

Grade ≥ 3 treatment-related adverse events (AEs) with incidence rates greater than 5% from the GEMSTONE-303 trial were considered in the analysis, comprising anemia (10.8% for sugemalimab plus CAPOX vs. 7.2% for CAPOX alone) and decreased white blood cell (6.6% vs. 3.0%), neutrophil (14.1% vs. 14.3%), and platelet counts (18.3% vs. 16.0%). Following clinical expert recommendations, decreased white blood cell and neutrophil counts were grouped as neutropenia, while anemia and a decreased platelet count were grouped as anemia, as these AEs are managed similarly in real-world Taiwanese clinical practice. To ensure a conservative analysis, the higher incidence rate within each category was used in the model (see Table 1).

### 2.6. Direct Medical Costs

Direct medical costs included medication, chemotherapy administration, non-medication services, supportive care during the PD state, terminal care in the 1 month preceding death, and the management of AEs. The four cost types in the PD state—supportive care, chemotherapy medication, chemotherapy administration, and terminal care costs—were mutually exclusive. Since sugemalimab has not yet been approved by Taiwan’s Food and Drug Administration, no local market price was available. Therefore, we used the price from China (CNY 12,375 per 600 mg, obtained from the Yaozh database on 5 March 2025 [37]), which was converted to U.S. dollars using the Taiwan Central Back 2024 exchange rate of 7.1975 CNY/USD (accessed on 28 July 2025 [43]), resulting in a hypothetical price of USD 1720 per 600 mg. All other costs were sourced from a CEA currently under review (see published abstract [26]), which examined the same patient population. These costs are reported in 2024 U.S. dollars (USD 1 = TWD 32.11) [44].

### 2.7. Health Utilities

Because the GEMSTONE-303 trial did not report utility values, we referenced the Australian Pharmaceutical Benefits Advisory Committee report for advanced G/GEJ patients treated with first-line CAPOX with and without nivolumab [38], considering sugemalimab’s similar mechanism as an immune checkpoint inhibitor [5]. The utility values were 0.812 and 0.798 (PF) and 0.746 and 0.721 (PD) for the intervention and comparator arms, respectively [38]. Disutilities for adverse events were obtained from published studies in advanced unresectable or metastatic esophageal squamous cell carcinoma [39] and non-small cell lung cancer [40], as data for G/GEJ adenocarcinoma were unavailable. All utility and disutility values were assumed to follow beta distributions (see Table 1).

### 2.8. Model Assumptions

Key model assumptions included the following: (1) patients received either sugemalimab plus CAPOX or CAPOX alone as first-line treatment, which continued until the earlier of either disease progression or the median treatment duration (6.3 months for sugemalimab plus CAPOX, 5.2 months for CAPOX alone) occurred in both the PD-L1 CPS ≥ 5 and ≥10 groups; (2) following progression, all patients received standardized supportive care; (3) only AEs with ≥5% incidence were modeled, and Grade 3/4 AEs incurred a utility decrement for the full cycle in which they occurred; (4) AEs were assumed to occur only during the first cycle in the PF state; (5) health states were assumed to be independent, consistent with a partitioned survival model structure; (6) in line with Taiwan’s NHIA, we modeled a point-based fee schedule, with one point valued at TWD 1 for medications and a conversion factor (assumed at TWD 0.9198 per point) for non-medication services; and (7) the direct medical costs for patients with advanced or metastatic gastric adenocarcinoma were assumed to be identical to those for patients with advanced or metastatic GEJ adenocarcinoma.

### 2.9. Sensitivity Analysis

Deterministic sensitivity analysis (DSA) and probabilistic sensitivity analysis (PSA) were performed to assess the impact of parameter uncertainty. The DSA varied each parameter within its 95% confidence interval or ±25% where data were unavailable. The PSA employed a Monte Carlo simulation with 5000 iterations, with results plotted on the cost-effectiveness plane and summarized using a cost-effectiveness acceptability curve. The expected value of perfect information (EVPI) was calculated to estimate the monetary value of eliminating parameter uncertainty.

### 2.10. Scenario Analysis

Several scenario analyses were performed to assess cost-effectiveness under varying assumptions: (1) substituting the equal value of life-years gained (evLYG) for QALYs; (2) altering treatment duration parameters, such as using the median PFS, continuing treatment until the protocol-defined maximum or until disease progression occurred; (3) varying the time horizon (5, 10, 20, and 30 years); (4) applying a gradual 10% reduction to the hypothesized price of sugemalimab; (5) assuming one point equals TWD 1 for all non-medication costs; and (6) fitting alternative parametric survival models, including log-normal models and best-fit models based solely on the AIC and BIC.

## 3. Results

### 3.1. Base-Case Analysis

In the base-case analysis, sugemalimab plus CAPOX provided 1.56 QALYs (equivalent to 2.05 life-years) at a direct medical cost of USD 123,373 (Table 2). In comparison, CAPOX alone yielded 1.17 QALYs (1.59 life-years) at a cost of USD 76,353. The addition of sugemalimab to CAPOX resulted in an incremental gain of 0.39 QALYs (0.46 life-years) and an incremental cost of USD 47,020. This corresponds to an ICER of USD 121,230 per QALY gained and an INMB of −USD 7478.

### 3.2. Base-Case Sensitivity Analysis

**Figure 1:**
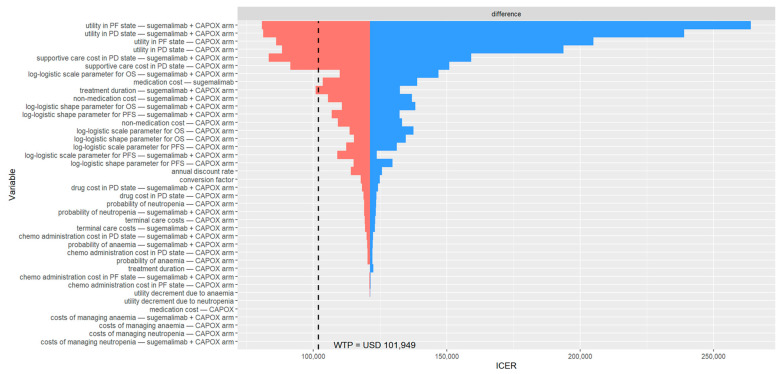
Deterministic sensitivity analysis results among patients with PD-L1 CPS ≥ 5. The black dashed line indicates the willingness-to-pay threshold of USD 101,949 per QALY gained. CAPOX, a combination of oxaliplatin and capecitabine; chemo, chemotherapy; ICER, incremental cost-effectiveness ratio; OS, overall survival; PD, progressed disease; PF, progression-free; PFS, progression-free survival; USD, United States dollar; WTP, willingness-to-pay.

**Figure 2:**
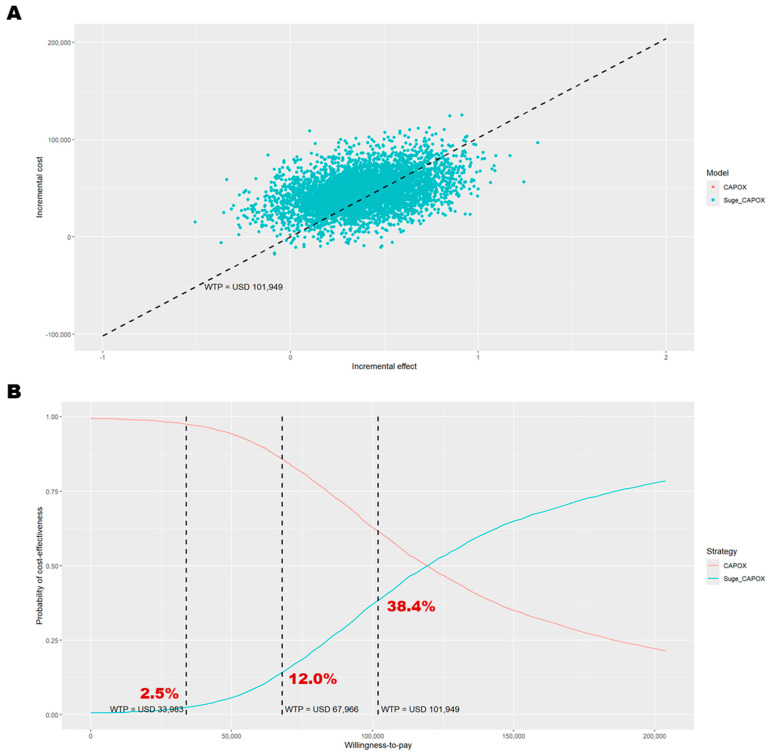
Probabilistic sensitivity analysis results among patients with PD-L1 CPS ≥ 5. (**A**) Outcomes over 5000 Monte Carlo simulations on the cost-effectiveness plane; (**B**) cost-effectiveness acceptance curve. In panel (**A**), the black dashed line represents the willingness-to-pay threshold of USD 101,949 per QALY gained. In panel (**B**), the dashed lines indicate willingness-to-pay thresholds of USD 33,983, USD 67,966, and USD 101,949 per QALY gained. CAPOX, a combination of oxaliplatin and capecitabine; Suge_CAPOX, sugemalimab plus CAPOX; WTP, willingness-to-pay.

The DSA identified the most influential parameters as the utility values for the PF and PD states, the cost of supportive care during the PD state, the medication cost of sugemalimab, and the treatment duration of sugemalimab plus CAPOX (Figure 1). At some extreme values for these parameters, the cost-effectiveness conclusion was reversed. In the PSA, 5000 Monte Carlo simulations indicated that most iterations fell within the northeast quadrant of the cost-effectiveness plane, reflecting greater effectiveness at higher costs for sugemalimab plus CAPOX (Figure 2). At a WTP threshold of three times the GDP per capita per QALY gained, the cost-effectiveness acceptability curve demonstrated only a 38.4% probability that the intervention would be considered cost-effective, with an EVPI of USD 6274 per person (Table 3).

### 3.3. Scenario Analysis

Most scenario analyses, except those varying health benefit measures, treatment duration, and the hypothesized price of sugemalimab, produced results consistent with the base-case analysis, with the probability of cost-effectiveness for the combination therapy ranging from 29.6% to 37.6% (Table 3). When evLYG was used as the health benefit measure, the combination of sugemalimab and CAPOX yielded an ICER of USD 101,801 per life-year gained, which is very close to the pre-defined WTP threshold of USD 101,949. Although these scenarios did not alter the overall cost-effectiveness conclusion, changes in treatment duration had a significant impact on the probability of the intervention being cost-effective. When first-line therapy was administered until the median PFS was reached, the probability of cost-effectiveness decreased to 31.2%. Extending the treatment duration to the earlier of the two, i.e., either the protocol-defined maximum was reached, or progression occurred, reduced the probability further to 8.5%, and administering treatment until progression lowered it to just 3.6%. In price reduction scenarios, a decrease of 28% in the cost of sugemalimab would be required to achieve cost-effectiveness, resulting in approximately USD 1204 per 600 mg.

### 3.4. Subgroup Analysis

**Figure A1:**
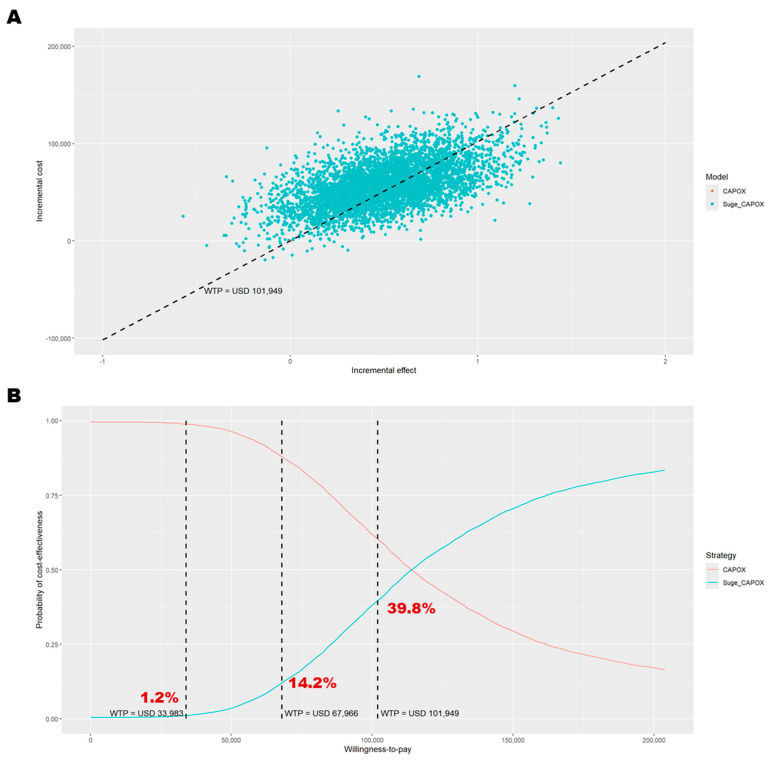
Probabilistic sensitivity analysis results among patients with PD-L1 CPS ≥ 10. (**A**) Outcomes over 5000 Monte Carlo simulations on the cost-effectiveness plane; (**B**) cost-effectiveness acceptance curve. In panel (**A**), the black dashed line represents the willingness-to-pay threshold of USD 101,949 per QALY gained. In panel (**B**), the dashed lines indicate willingness-to-pay thresholds of USD 33,983, USD 67,966, and USD 101,949 per QALY gained. CAPOX, a combination of oxaliplatin and capecitabine; Suge_CAPOX, sugemalimab plus CAPOX; WTP, willingness-to-pay.

**Figure A2:**
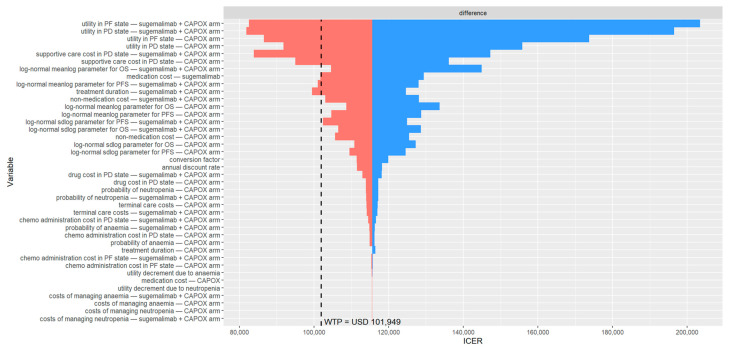
Deterministic sensitivity analysis results among patients with PD-L1 CPS ≥ 10. The black dashed line indicates the willingness to pay threshold of USD 101,949 per QALY gained. CAPOX, a combination of oxaliplatin and capecitabine; chemo, chemotherapy; ICER, incremental cost-effectiveness ratio; OS, overall survival; PD, progressed disease; PF, progression-free; PFS, progression-free survival; USD, United States dollar; WTP, willingness-to-pay.

In patients with a PD-L1 CPS ≥ 10, who derived greater clinical benefit from the addition of sugemalimab to CAPOX, the cost-effectiveness conclusions were consistent with those observed in the base-case analysis (PD-L1 CPS ≥ 5). Sugemalimab plus CAPOX provided 1.65 QALYs (equivalent to 2.17 life-years) at a direct medical cost of USD 130,008 (Table 2), whereas CAPOX alone yielded 1.15 QALYs (1.56 life-years) at a cost of USD 71,990. Thus, adding sugemalimab to CAPOX resulted in an incremental gain of 0.5 QALYs (0.61 life-years) and an additional cost of USD 58,017. This translates to an ICER of USD 115,568 per QALY gained and an INMB of −USD 6837. The PSA indicated a similar probability of cost-effectiveness (39.8%, Figure A1). The most influential parameters in the DSA remained consistent with those identified for patients with PD-L1 CPS ≥ 5 (Figure A2). The scenario analyses confirmed that treatment duration substantially affects the probability of sugemalimab plus CAPOX being cost-effective (ranging from 4.2% to 32.8%, Table A3). A price reduction of 25% for sugemalimab would be required for the combination to become cost-effective, approximately USD 1376 per 600 mg. When the evLYG was used as the health benefit measure, the probability of cost-effectiveness increased to 60.5%.

## 4. Discussion

In our base-case analysis, the combination of sugemalimab and CAPOX was not cost-effective as a first-line treatment for advanced or metastatic G/GEJ adenocarcinoma in patients with either a PD-L1 CPS ≥ 5 or ≥10. These findings align with two recent studies conducted from the Chinese healthcare perspective [23,24]. When the evLYG was used as the health benefit measure, sugemalimab approached borderline cost-effectiveness for PD-L1 CPSs ≥ 5 and was cost-effective when the score was ≥10. Price scenario analyses indicated that a reduction of approximately 20–30% from the hypothesized price, equivalent to about USD 1204 per 600 mg (for PD-L1 CPS ≥ 5) or USD 1376 per 600 mg (for PD-L1 CPS ≥ 10), would be required for sugemalimab to achieve cost-effectiveness, although considerable uncertainty remains. While other scenario analyses also yielded unfavorable cost-effectiveness results, assumptions regarding treatment duration had a particularly substantial impact on the outcomes.

The probability of sugemalimab being cost-effective decreased markedly as the assumed treatment duration extended. In our base-case model, which used the median treatment duration from the GEMSTONE-303 trial, the probability of cost-effectiveness was 38.4% and 39.8% for patients with CPSs ≥ 5 and ≥10, respectively. When first-line therapy was modeled until median PFS was reached, the earlier of two occurrences, i.e., the protocol-defined maximum was reached or progression occurred, and disease progression, the probabilities of sugemalimab being cost-effective among PD-L1 CPS ≥ 5 patients declined to 31.2%, 8.5%, and 3.6%, respectively. We conducted three additional price scenario analyses based on different treatment durations. If the NHIA reimbursed sugemalimab based on the median PFS (7.72 months), the price would need to be reduced by approximately 35–38% to achieve cost-effectiveness (PD-L1 CPS ≥ 5: USD 1066 per 600 mg; PD-L1 CPS ≥ 10: USD 1117 per 600 mg); if reimbursement was based on the earlier of either the protocol-defined maximum being reached (24 months for sugemalimab or placebo, 4.5 months for CAPOX) or progression occurring, a price reduction of about 62% would be necessary (USD 653 per 600 mg); and if reimbursement continued until disease progression, a reduction of approximately 68% would be required (USD 550 per 600 mg). These findings highlight the significant impact of treatment duration assumptions.

To simulate long-term cost-effectiveness outcomes, it is necessary to make assumptions regarding decision-analytical models and key parameters in model-based health technology assessments. Although our base-case analysis reached similar conclusions to a recently published Chinese study [23], there was a substantial difference in the estimated probability of sugemalimab plus CAPOX being cost-effective. Tang et al. reported a 0% probability of cost-effectiveness for the sugemalimab regimen [23], whereas our base-case analysis indicated a 38.4% probability. Most of this discrepancy arises from differences in utility values, treatment duration assumptions, and time horizons. Tang et al. used utility data from patients with HER2-positive advanced gastric cancer and assumed treatment continued until disease progression, with a 10-year simulation horizon [23]. In contrast, we sourced utility values from patients with HER2-negative advanced gastric cancer, which we believe better reflects our study population. We also assumed treatment duration was equal to the median duration observed in the GEMSTONE-303 trial, with a 40-year lifetime horizon. When we applied a 10-year time horizon, the probability of sugemalimab plus CAPOX being cost-effective decreased slightly to 35.4%, and using the same utility values as Tang et al. led to a dramatic reduction in cost-effectiveness probability to 12.1%. Further aligning our treatment duration assumptions with theirs (i.e., treatment until disease progression) resulted in a substantial decrease to 0.06%, closely matching their findings. Although there is no universal standard for parameter selection in health technology assessments, since these choices are context-specific, transparency in methodology and assumptions is essential to inform sound decision-making.

This study shares limitations common to model-based oncology cost-effectiveness analyses, primarily due to data inaccessibility [26,27,28,29]. Without patient-level trial data, we could not conduct survival modeling that accurately reflects transition probabilities, limiting our ability to simulate with a Markov model and evaluate model uncertainty. We were also unable to estimate treatment heterogeneity or address more nuanced equity considerations. While using a parametric distribution to model the time to treatment discontinuation would be preferable, we lacked access to individual patient data from GEMSTONE-303 and therefore, assumed the treatment duration to be equal to the median duration reported, treating it as a fixed parameter in the PSA. Additionally, in the absence of detailed information on AE timing, we assumed that all AEs occurred in the first treatment cycle, a common approach in oncology cost-effectiveness studies. Because advanced gastric cancer-specific disutility values for AEs were unavailable, we relied on estimates from other cancer types, which may introduce additional uncertainty. Direct medical costs were obtained from a previous study that used NHI claims data for estimation; however, the sample may not fully represent the trial population because the NHI claims data lack laboratory examination results and diagnostic codes for GEJ adenocarcinoma. Additionally, the exclusion of self-financed medical expenses from the NHI database may have led to a misclassification of treatment groups, potentially affecting cost estimates. Anemia and thrombocytopenia are managed differently in clinical practice; however, we grouped these AEs based on clinical oncologist judgment, as distinguishing between them using Taiwan’s NHI claims data is extremely challenging. This approach may underestimate heterogeneity and increase uncertainty in our cost-effectiveness model.

There are two directions for future research. First, a health technology reassessment should be conducted once sufficient real-world data have been collected. Second, head-to-head cost-effectiveness comparisons among immune checkpoint inhibitors for the treatment of advanced or metastatic gastric cancer are warranted, particularly in countries where these agents are not yet available as first-line treatment options.

## 5. Conclusions

The combination of sugemalimab and CAPOX is not cost-effective for patients with advanced or metastatic G/GEJ adenocarcinoma from the perspective of Taiwan’s NHIA compared to CAPOX alone. To achieve cost-effectiveness, the hypothesized price of sugemalimab would need to be reduced by 20–30% (to approximately USD 1204 per 600 mg for PD-L1 CPS ≥ 5 and USD 1376 per 600 mg for PD-L1 CPS ≥ 10), assuming first-line therapy is administered for the median treatment duration observed in the GEMSTONE-303 trial. If reimbursement continued until disease progression, a reduction of approximately 68% would be required (USD 550 per 600 mg).

## Figures and Tables

**Table 1:** Model parameters, baseline values, ranges, and distributions for sensitivity analyses.

Parameters		Base-Case Analysis	One-Way Sensitivity Analysis		Probabilistic Sensitivity Analysis			Source
		Value	Range (95% CI or ±25%)		Distribution	Parameter 1	Parameter 2	
Overall survival (base-case)								
PD-L1 CPS ≥ 5								
Sugemalimab + CAPOX								[17]
Log-logistic	Shape	1.904	1.667	2.174	normal (mean, se)	1.904	0.129	
	Scale	16.860	14.921	19.052	normal (mean, se)	16.860	1.051	
CAPOX								[17]
Log-logistic	Shape	2.004	1.772	2.266	normal (mean, se)	2.004	0.126	
	Scale	13.331	11.894	14.942	normal (mean, se)	13.331	0.776	
PD-L1 CPS ≥ 10								
Sugemalimab + CAPOX								
Log-normal	Mean on log scale	2.912	2.735	3.089	normal (mean, se)	2.912	0.090	[17]
	SD on log scale	0.922	0.783	1.087	normal (mean, se)	0.922	0.077	
CAPOX								
Log-normal	Mean on log scale	2.538	2.363	2.712	normal (mean, se)	2.538	0.089	[17]
	SD on log scale	0.947	0.818	1.098	normal (mean, se)	0.947	0.071	
Progression-free survival (base-case)								
PD-L1 CPS ≥ 5								
Sugemalimab + CAPOX								[17]
Log-logistic	Shape	2.212	1.936	2.528	normal (mean, se)	2.212	0.15	
	Scale	9.015	8.082	10.056	normal (mean, se)	9.015	0.503	
CAPOX								[17]
Log-logistic	Shape	2.445	2.156	2.772	normal (mean, se)	2.445	0.157	
	Scale	7.305	6.629	8.05	normal (mean, se)	7.305	0.362	
PD-L1 CPS ≥ 10								
Sugemalimab + CAPOX								
Log-normal	Mean on log scale	2.278	2.123	2.433	normal (mean, se)	2.278	0.079	[17]
	SD on log scale	0.776	0.656	0.917	normal (mean, se)	0.776	0.066	
CAPOX								
Log-normal	Mean on log scale	2.034	1.884	2.183	normal (mean, se)	2.034	0.077	[17]
	SD on log scale	0.777	0.666	0.906	normal (mean, se)	0.777	0.061	
Overall survival (scenario)								
PD-L1 CPS ≥ 5								
Sugemalimab + CAPOX								[17]
Log-normal	Mean on log scale	2.838	2.708	2.967	normal (mean, se)	2.838	0.066	
	SD on log scale	0.921	0.818	1.037	normal (mean, se)	0.921	0.056	
CAPOX								[17]
Log-normal	Mean on log scale	2.593	2.476	2.709	normal (mean, se)	2.593	0.060	
	SD on log scale	0.862	0.773	0.961	normal (mean, se)	0.862	0.048	
PD-L1 CPS ≥ 10								
Sugemalimab + CAPOX								
Log-logistic	Shape	1.870	1.557	2.246	normal (mean, se)	1.870	0.175	[17]
	Scale	18.193	15.352	21.559	normal (mean, se)	18.193	1.576	
CAPOX								
Log-logistic	Shape	1.840	1.555	2.177	normal (mean, se)	1.840	0.158	[17]
	Scale	12.808	10.823	15.159	normal (mean, se)	12.808	1.101	
Progression-free survival (scenario)								
PD-L1 CPS ≥ 5								
Sugemalimab + CAPOX								[17]
Log-normal	Mean on log scale	2.224	2.111	2.337	normal (mean, se)	2.224	0.058	
	SD on log scale	0.785	0.698	0.884	normal (mean, se)	0.785	0.048	
CAPOX								[17]
Log-normal	Mean on log scale	2.005	1.905	2.105	normal (mean, se)	2.005	0.051	
	SD on log scale	0.715	0.640	0.798	normal (mean, se)	0.715	0.040	
PD-L1 CPS ≥ 10								
Sugemalimab + CAPOX								
Log-logistic	Shape	2.193	1.820	2.642	normal (mean, se)	2.193	0.208	[17]
	Scale	9.503	8.156	11.072	normal (mean, se)	9.503	0.741	
CAPOX								
Log-logistic	Shape	2.227	1.87	2.652	normal (mean, se)	2.227	0.198	[17]
	Scale	7.604	6.567	8.804	normal (mean, se)	7.604	0.569	
Median treatment duration (months)								
Sugemalimab + CAPOX		6.3						[17]
CAPOX		5.2						[17]
Medication cost (per year, USD)								
Sugemalimab		59,814	44,860	74,767	fixed			[37]
CAPOX		8959	8474	9444	gamma (μ, s)	8959	247	[26]
Chemo administration cost (per year, USD)		1226	1148	1303	gamma (μ, s)	1226	39	[26]
Non-medication cost (per year, USD)		25,925	22,402	29,449	gamma (μ, s)	25,925	1798	[26]
Supportive care cost (per year, USD)		63,077	41,234	84,920	gamma (μ, s)	63,077	11,144	[26]
PD drug cost (per year, USD)		4682	3878	5485	gamma (μ, s)	4682	410	[26]
PD chemo administration cost (per year, USD)		2018	1551	2485	gamma (μ, s)	2018	238	[26]
Terminal care costs (per year, USD)		54,893	50,519	59,266	gamma (μ, s)	54,893	2231	[26]
Adverse event cost (per year, USD)								
Neutropenia		769	388	1151	gamma (μ, s)	769	195	[26]
Anemia		1494	959	2029	gamma (μ, s)	1494	273	[26]
Adverse event probability								
Sugemalimab + CAPOX								
Neutropenia		0.141	0.10575	0.17625	fixed			[17]
Anemia		0.183	0.13725	0.22875	fixed			[17]
CAPOX								
Neutropenia		0.143	0.10725	0.17875	fixed			[17]
Anemia		0.16	0.12	0.2	fixed			[17]
Utility								
Sugemalimab (PF state)		0.812	0.609	1	beta (α, β)	10.74	2.49	[38]
Sugemalimab (PD state)		0.746	0.5595	0.9325	beta (α, β)	14.87	5.06	[38]
Chemotherapy (PF state)		0.798	0.5985	0.9975	beta (α, β)	11.62	2.94	[38]
Chemotherapy (PD state)		0.721	0.54075	0.90125	beta (α, β)	16.43	6.36	[38]
Disutility								
Neutropenia		0.2	0.15	0.25	beta (α, β)	48.97	195.89	[39]
Anemia		0.07	0.0525	0.0875	beta (α, β)	57.09	758.52	[40]
Annual discount rate		0.03	0	0.05	fixed			[41]
Conversion factor		0.9198	0.853	0.9913	uniform (min, max)	0.853	0.9913	[42]

All costs are listed in 2024 U.S. dollars, using the average 2024 exchange rate between the U.S. dollar and the New Taiwan dollar (USD 1 = TWD 32.11). AE, adverse event; CAPOX, combination of oxaliplatin and capecitabine; CI, confidence interval; CPS, combined positive score; max, maximum; NHIRD, National Health Insurance Research Database; PD, progressed disease; PD-L1, programmed cell death-ligand 1; PF, progression-free; SD, standard deviation; SE, standard error.

**Table 2:** Base-case results.

	Outcomes of Partitioned Survival Models				Incremental Changes	
Population	PD-L1 CPS ≥ 5		PD-L1 CPS ≥ 10		PD-L1 CPS ≥ 5	PD-L1 CPS ≥ 10
Treatment Strategy	Sugemalimab + CAPOX	CAPOX	Sugemalimab + CAPOX	CAPOX	Sugemalimab + CAPOX vs. CAPOX	Sugemalimab + CAPOX vs. CAPOX
Cost (USD)	123,373	76,353	130,008	71,990	47,020	58,018
Total cost of PF state	55,015	21,951	56,316	23,068	33,064	33,248
Medication cost	30,264	2846	30,741	2830	27,418	27,911
Non-medication cost	24,751	19,105	25,575	20,238	5646	5337
Total cost of PD state	68,358	54,401	73,692	48,922	13,957	24,770
Medication cost	4740	3726	5127	3329	1014	1798
Non-medication cost	63,618	50,675	68,565	45,593	12,943	22,972
LY	2.05	1.59	2.17	1.56	0.46	0.61
PF state	1.03	0.79	1.06	0.84	0.24	0.22
PD state	1.02	0.80	1.11	0.72	0.22	0.39
QALY	1.56	1.17	1.65	1.15	0.39	0.50
PF state	0.80	0.59	0.83	0.64	0.21	0.19
PD state	0.76	0.58	0.82	0.51	0.18	0.31
ICERs						
Incremental cost per LY gained					102,559	94,385
Incremental cost per QALY gained					121,230	115,568
INMB						
LY					−279	4648
QALY					−7478	−6837
EVPI/person					6274	6993

All costs are listed in 2024 U.S. dollars, using the average 2024 exchange rate between the U.S. dollar and the New Taiwan dollar (USD 1 = TWD 32.11). CAPOX, a combination of oxaliplatin and capecitabine; CPS, combined positive score; EVPI, expected value of perfect information; ICER, incremental cost-effectiveness ratio; INMB, incremental net monetary benefit; LY, life-year; PD, progressed disease; PD-L1, programmed cell death-ligand 1; PF, progression-free; QALY, quality-adjusted life-year.

**Table 3:** Scenario analyses with base-case and probabilistic sensitivity analyses for sugemalimab plus CAPOX versus CAPOX alone among patients with PD-L1 CPS ≥ 5.

Scenario	Sugemalimab + CAPOX vs. Placebo + CAPOX							
	Base-Case Analysis				Probabilistic Sensitivity Analysis			
	Incremental Costs (USD)	Incremental Effectiveness (QALYs)	ICER	INMB (USD)	ICER	INMB (USD)	Probability of Cost-Effectiveness	EVPI/Person
0. Base case	47,020	0.39	121,230	−7478	119,409	−6837	38.4%	6274
1. Effectiveness measure: Equal value of life-years gained	47,020	0.46	102,559	−279	101,801	68	49.7%	6325
2a. Treatment duration: Median PFS (7.72 months vs. 6.24 months)	51,380	0.39	132,470	−11,838	130,523	−11,189	31.2%	4748
2b. Treatment duration: Protocol-defined maximum	72,100	0.39	185,891	−32,558	183,604	−31,975	8.5%	873
2c. Treatment duration: Administered until disease progressed	81,444	0.39	209,982	−41,902	208,520	−41,731	3.6%	303
3a. Time horizons: 5 years	39,352	0.28	140,143	−10,725	138,879	−10,379	29.6%	3762
3b. Time horizons: 10 years	43,824	0.34	127,221	−8705	125,669	−8206	35.4%	5236
3c. Time horizons: 20 years	46,115	0.37	122,706	−7801	120,980	−7206	37.7%	5986
3d. Time horizons: 30 years	46,762	0.38	121,632	−7567	119,842	−6940	38.1%	6193
4a. Price: 90% of the hypothesized price of sugemalimab	44,282	0.39	114,171	−4740	112,428	−4103	43.0%	7384
4b. Price: 80% of the hypothesized price of sugemalimab	41,545	0.39	107,112	−2002	105,447	−1370	47.7%	8622
4c. Price: 70% of the hypothesized price of sugemalimab	38,807	0.39	100,054	734	98,465	1364	52.2%	8626
4d. Price: 60% of the hypothesized price of sugemalimab	36,069	0.39	92,995	3472	91,484	4098	57.0%	7388
4e. Price: 50% of the hypothesized price of sugemalimab	33,331	0.39	85,936	6210	84,503	6831	61.6%	6277
5. Conversion factor: One point equals TWD 1	48,645	0.39	125,418	−9102	123,542	−8456	36.6%	5919
6a. Survival models: Log-logistic	49,103	0.41	120,171	−7445	118,633	−6879	37.5%	5975
6b. Best-fit survival models based solely on AIC and BIC	51,384	0.43	119,602	−7584	118,180	−7071	37.6%	6028

AIC, Akaike information criterion; BIC, Bayesian information criterion; CPS, combined positive score; EVPI, expected value of perfect information; ICER, incremental cost-effectiveness ratio; INMB, incremental net monetary benefit; PFS, progression-free survival; TWD, New Taiwan dollar; PD-L1, programmed death-ligand 1; QALYs, quality-adjusted life-years.

## Data Availability

The reconstructed pseudo-individual patient data used for effectiveness extrapolation and model simulation will be made available by the authors upon request.

## Appendix A

**Table A1:** CHEERS checklist.

Topic	No.	Item	Location Where Item Is Reported
**Title**			
	1	Identify the study as an economic evaluation and specify the interventions being compared.	Title page
**Abstract**			
	2	Provide a structured summary that highlights context, key methods, results, and alternative analyses.	Abstract section
**Introduction**			
**Background and objectives**	3	Give the context for the study, the study question, and its practical relevance for decision making in policy or practice.	Introduction section
**Methods**			
**Health economic analysis plan**	4	Indicate whether a health economic analysis plan was developed and where available.	Health economic analysis plan subsection
**Study population**	5	Describe characteristics of the study population (such as age range, demographics, socioeconomic, or clinical characteristics).	Study population subsection
**Setting and location**	6	Provide relevant contextual information that may influence findings.	Health economic analysis plan subsection
**Comparators**	7	Describe the interventions or strategies being compared and why chosen.	Intervention and comparator subsection
**Perspective**	8	State the perspective(s) adopted by the study and why chosen.	Health economic analysis plan subsection
**Time horizon**	9	State the time horizon for the study and why appropriate.	Health economic analysis plan subsection
**Discount rate**	10	Report the discount rate(s) and reason chosen.	Health economic analysis plan subsection
**Selection of outcomes**	11	Describe what outcomes were used as the measure(s) of benefit(s) and harm(s).	Health economic analysis plan subsection
**Measurement of outcomes**	12	Describe how outcomes used to capture benefit(s) and harm(s) were measured.	Clinical efficacy, Treatment-Related adverse events, and Health utilities subsections
**Valuation of outcomes**	13	Describe the population and methods used to measure and value outcomes.	Health utilities subsection
**Measurement and valuation of resources and costs**	14	Describe how costs were valued.	Direct medical costs subsection
**Currency, price date, and conversion**	15	Report the dates of the estimated resource quantities and unit costs, plus the currency and year of conversion.	Health economic analysis plan and Direct medical costs subsections
**Rationale and description of model**	16	If modelling is used, describe in detail and why used. Report if the model is publicly available and where it can be accessed.	Health economic analysis plan and Clinical efficacy subsections
**Analytics and assumptions**	17	Describe any methods for analysing or statistically transforming data, any extrapolation methods, and approaches for validating any model used.	Model assumptions subsection
**Characterising heterogeneity**	18	Describe any methods used for estimating how the results of the study vary for subgroups.	Health economic analysis plan subsection
**Characterising distributional effects**	19	Describe how impacts are distributed across different individuals or adjustments made to reflect priority populations.	Not available
**Characterising uncertainty**	20	Describe methods to characterise any sources of uncertainty in the analysis.	Sensitivity analysis and Scenario analysis subsections
**Approach to engagement with patients and others affected by the study**	21	Describe any approaches to engage patients or service recipients, the general public, communities, or stakeholders (such as clinicians or payers) in the design of the study.	Not available
**Results**			
**Study parameters**	22	Report all analytic inputs (such as values, ranges, references) including uncertainty or distributional assumptions.	Methods section and Table 1
**Summary of main results**	23	Report the mean values for the main categories of costs and outcomes of interest and summarise them in the most appropriate overall measure.	Base-Case analysis and Base-Case sensitivity analysis subsections
**Effect of uncertainty**	24	Describe how uncertainty about analytic judgments, inputs, or projections affect findings. Report the effect of choice of discount rate and time horizon, if applicable.	Base-Case sensitivity analysis, Scenario analysis, and Subgroup analysis subsections
**Effect of engagement with patients and others affected by the study**	25	Report on any difference patient/service recipient, general public, community, or stakeholder involvement made to the approach or findings of the study	Not available
**Discussion**			
**Study findings, limitations, generalisability, and current knowledge**	26	Report key findings, limitations, ethical or equity considerations not captured, and how these could affect patients, policy, or practice.	Discussion section
**Other relevant information**			
**Source of funding**	27	Describe how the study was funded and any role of the funder in the identification, design, conduct, and reporting of the analysis	Funding section
**Conflicts of interest**	28	Report authors’ conflicts of interest according to journal or International Committee of Medical Journal Editors requirements.	Conflict of interest section

**Table A2:** AIC and BIC for each parametric model.

Regimen	Distribution	Progression-Free Survival		Overall Survival	
		AIC	BIC	AIC	BIC
**PD-L1 CPS ≥ 5**					
Sugemalimab + CAPOX	Exponential	1068.252	1071.737	1304.787	1308.272
	Weibull	1040.966	1047.936	1286.684	1293.653
	Gompertz	1067.179	1074.148	1303.422	1310.392
	Log-normal	1006.989	1013.959	1269.906	1276.875
	Log-logistic	1008.241	1015.210	1268.815	1275.784
	Generalized gamma	1012.230	1022.685	1272.836	1283.290
	Gamma	1027.590	1034.560	1280.048	1287.018
Placebo + CAPOX	Exponential	1107.486	1110.958	1386.945	1390.418
	Weibull	1064.901	1071.846	1363.771	1370.716
	Gompertz	1100.335	1107.279	1383.564	1390.508
	Log-normal	1031.080	1038.025	1343.358	1350.303
	Log-logistic	1031.023	1037.968	1344.653	1351.597
	Generalized gamma	1031.948	1042.365	1345.268	1355.685
	Gamma	1048.522	1055.467	1355.548	1362.492
**PD-L1 CPS ≥ 10**					
Sugemalimab + CAPOX	Exponential	556.6350	559.5025	700.3561	703.2237
	Weibull	543.0814	548.8165	691.7390	697.4740
	Gompertz	556.9605	562.6955	700.5508	706.2858
	Log-normal	523.6356	529.3707	681.7808	687.5159
	Log-logistic	526.4785	532.2136	682.9770	688.7121
	Generalized gamma	527.9641	536.5668	684.7373	693.3399
	Gamma	535.8626	541.5977	688.2621	693.9972
Placebo + CAPOX	Exponential	580.5273	583.3794	746.9172	749.7692
	Weibull	564.0427	569.7467	740.0807	745.7848
	Gompertz	579.3337	585.0377	747.3778	753.0819
	Log-normal	549.3813	555.0854	733.1846	738.8886
	Log-logistic	550.6078	556.3118	733.3101	739.0141
	Generalized gamma	550.9119	559.4680	735.0102	743.5663
	Gamma	557.4220	563.1261	737.2156	742.9196

AIC, Akaike information criterion; BIC, Bayesian information criterion; CAPOX, combination of capecitabine and oxaliplatin; CPS, combined positive score; PD-L1, programmed cell death-ligand 1.

**Table A3:** Scenario analyses with base-case and probabilistic sensitivity analyses for sugemalimab plus CAPOX versus CAPOX alone among patients with PD-L1 CPS ≥ 10.

Scenario	Sugemalimab + CAPOX vs. Placebo + CAPOX							
	Base-Case Analysis				Probabilistic Sensitivity Analysis			
	Incremental Costs (USD)	Incremental Effectiveness (QALYs)	ICER	INMB (USD)	ICER	INMB (USD)	Probability of Cost-Effectiveness	EVPI/Person
0. Base case	58,017	0.50	115,568	−6837	113,965	−6098	39.8%	6993
1. Effectiveness measure: Equal value of life-years gained	58,017	0.61	94,385	4648	93,893	4952	60.5%	5024
2a. Treatment duration: Median PFS (7.92 months vs. 6.93 months)	62,572	0.50	124,641	−11,392	122,928	−10,647	32.8%	5336
2b. Treatment duration: Protocol-defined maximum	86,230	0.50	171,766	−35,049	169,454	−34,259	7.9%	854
2c. Treatment duration: Administered until disease progressed	94,078	0.50	187,400	−42,898	185,641	−42,474	4.2%	362
3a. Time horizons: 5 years	48,985	0.39	125,418	−9166	124,393	−8786	34.1%	4764
3b. Time horizons: 10 years	55,509	0.47	117,607	−7390	116,260	−6780	38.4%	6383
3c. Time horizons: 20 years	57,671	0.49	115,824	−6908	114,292	−6199	39.7%	6901
3d. Time horizons: 30 years	57,957	0.50	115,611	−6849	114,026	−6117	39.7%	6975
4a. Price: 90% of the hypothesized price of sugemalimab	55,235	0.50	110,026	−4054	108,490	−3320	44.4%	8158
4b. Price: 80% of the hypothesized price of sugemalimab	52,453	0.50	104,484	−1272	103,016	−541	49.4%	9465
4c. Price: 70% of the hypothesized price of sugemalimab	49,671	0.50	98,943	1509	97,541	2237	54.0%	8663
4d. Price: 60% of the hypothesized price of sugemalimab	46,889	0.50	93,401	4291	92,066	5015	58.5%	7448
4e. Price: 50% of the hypothesized price of sugemalimab	44,107	0.50	87,859	7073	86,591	7794	62.9%	6354
5. Conversion factor: One point equals TWD 1	60,490	0.50	120,494	−9310	118,856	−8580	36.2%	6268
6a. Survival models: Log-logistic	56,523	0.48	116,472	−7048	114,450	−6142	40.2%	7469

CPS, combined positive score; EVPI, expected value of perfect information; ICER, incremental cost-effectiveness ratio; INMB, incremental net monetary benefit; PFS, progression-free survival; TWD, New Taiwan dollar; PD-L1, programmed death-ligand 1; QALYs, quality-adjusted life-years.
